# Alcohol Consumption and Risk of Type 2 Diabetes in East Asian Populations: Do Healthy Patterns of Consumption Exist?

**DOI:** 10.2188/jea.JE20170151

**Published:** 2018-03-05

**Authors:** Rob M. van Dam

**Affiliations:** Saw Swee Hock School of Public Health, Department of Medicine, Yong Loo Lin School of Medicine, National University of Singapore and National University Health System, Republic of Singapore

Excessive alcohol consumption is a major cause of ill health worldwide.^1^ However, results from various studies have suggested that modest alcohol consumption can lower risk of type 2 diabetes and coronary artery disease.^1^^,^^2^ The relationship between alcohol consumption and chronic disease risk appears to be complex, not only depending on the specific disease outcome, but also on the pattern of alcohol consumption. The same amount of alcohol consumed over a certain period is likely to have a different effect for binge drinking as compared with lower but regular consumption. Furthermore, the effect of alcohol intake is likely to be modified by sex, with possible beneficial effects of moderate alcohol consumption on risk of type 2 diabetes restricted to women.^2^ In addition, common variants in the alcohol dehydrogenase (ADH) and aldehyde dehydrogenase (ALDH) gene affect the activity of enzymes that metabolize alcohol.^3^ In persons of East Asian ancestry, genetic variants that lead to slower metabolism of alcohol and its metabolite acetaldehyde are much more common than in persons of European ancestry.^4^ Thus, the impact of alcohol consumption on the development of type 2 diabetes is likely to depend on patterns of alcohol consumption, sex, and ethnicity.^5^

In this issue of the *Journal of Epidemiology*, Jisun Lim et al report an association between alcohol consumption and the prevalence of impaired fasting glucose (IFG) and undiagnosed diabetes in the Korea National Health and Nutrition Examination Survey (KNHANES).^6^ This study had a cross-sectional design, but reverse causation seems unlikely, as participants with known diabetes were excluded. The study had several strengths. First, the authors considered patterns of alcohol consumption by examining the frequency of alcohol consumption in relation to diabetes separately for persons with a high-risk level of consumption and participants with lower levels of consumption. Second, instead of combining all participants with no current alcohol consumption as the reference group, they distinguished between never consumers, past consumers, and participants with a very low frequency of consumption (<1/month) and used the latter as the reference group. This is relevant, because past alcohol consumers are likely to include individuals who quit consumption because of ill health, thus making them inappropriate as a reference group. In fact, a recent meta-analysis only showed an association between moderate alcohol consumption and a lower diabetes risk in studies using all non-current consumers as a reference group and not in studies using never consumers as the reference group.^2^ The study by Lim et al is a valuable addition to the literature because only a limited number of studies attempted to distinguish frequency and level of consumption or used a reference group not including past alcohol consumers.

Lim et al report that a high-risk level of consumption was significantly associated with a higher prevalence of IFG in both men and women and a higher prevalence of diabetes in men. This association was observed even for high-risk drinking with a frequency of only 2 to 4 times per month. High-risk drinking was defined as at least 7 drinks per sitting in men and at least 5 drinks per sitting in women. Thus, the amount of alcohol consumed at a single sitting was very high, but the average daily alcohol intake for a frequency of 2 to 4 times per month was only 8 g in women and 12 g in men. These results suggest that binge drinking may be associated with a higher risk of type 2 diabetes, even in the absence of high average daily consumption. Furthermore, high-frequency binge drinking was more strongly associated with IFG and diabetes risk than low-frequency binge drinking.

For modest levels of alcohol intake, regular drinking (on most or all days of the week) was associated with a lower diabetes prevalence in women as compared with rare consumption. The average alcohol consumption in this group was 17 grams per day corresponding with about 1.5 standard alcoholic drinks. These results should be interpreted with caution, as this consumption pattern was not associated with prevalence of IFG and no trend for increasing frequency of consumption was observed suggesting that this association might be due to chance or residual confounding. However, these results do fit into the overall evidence from meta-analyses of prospective cohort studies and short term randomized trials. In prospective cohort studies, moderate alcohol consumption was associated with a lower risk of type 2 diabetes in women but not in men.^2^ Similarly, moderate alcohol consumption as compared with non-consumption in trials reduced fasting plasma insulin concentrations in women only, suggesting a sex-specific beneficial effect on insulin sensitivity.^7^ It has been postulated that the sex difference in the association between alcohol consumption and diabetes risk may be due to the impact of alcohol on sex hormones.^2^ Alcohol lowers testosterone levels, which are associated with a higher diabetes risk in women but with a lower diabetes risk in men.

In men participating in KNAHES, even frequent *low-risk* drinking, with an average intake of 27 grams per day, was associated with higher risk of diabetes. In previous studies, very high total alcohol consumption (>60 g in men and >50 g in women per day) was associated with a higher diabetes risk.^2^ The higher prevalence of variants in alcohol metabolizing genes that can lead to higher circulating concentrations of alcohol and acetaldehyde for the same level of alcohol intake may increase diabetes risk at a lower level of consumption in East Asian populations.^4^ Interestingly, a Mendelian randomization study in a Korean population also suggested that moderate alcohol consumption increases fasting blood glucose concentrations in men.^4^ This study used ALDH gene variants as an instrumental variable reflecting alcohol consumption. Mendelian randomization has been suggested to reflect causal relationships, potentially avoiding confounding and reverse causation that can affect traditional epidemiological approaches. However, in the case of alcohol consumption, this approach may have limitations, as the genetic instrument cannot clearly distinguish patterns of alcohol consumption and reflects not only levels of consumption but also differences in alcohol metabolism that may affect diabetes risk.

In sum, the study by Lim et al suggests that binge drinking has adverse effects on the development of type 2 diabetes. The high prevalence of binge drinking reported in this population of adult Koreans over 30 years of age is alarming. Regular consumption of moderate amounts of alcohol consumption may lower risk of type 2 diabetes in women. However, this level of consumption can still increase risk of traffic accidents, future transition to alcoholism, and several common forms of cancer, including common cancers such as breast and colorectal cancer.^1^ In light of these health risks associated with alcohol consumption, recent national guidelines recommend consumption of a maximum of 14 standard alcoholic drinks per week in the United Kingdom^8^ and a maximum of one drink per day in the Netherlands.^9^ In East Asian populations, high alcohol consumption may be of even greater public health concern because a large proportion of the population is genetically predisposed to high levels of the carcinogenic alcohol metabolite acetaldehyde.^3^^,^^4^ Thus, light-to-moderate regular consumption of alcohol may be a beneficial consumption pattern for women for the prevention of diabetes, but even for this pattern of consumption, the overall health impact is likely to be negative for many individuals.

## References

[1] Rehm J, Gmel GE Sr, Gmel G, The relationship between different dimensions of alcohol use and the burden of disease—an update. Addiction. 2017 Jun;112(6):968–1001. 10.1111/add.13757 28220587 PMC5434904

[2] Knott C, Bell S, Britton A Alcohol consumption and the risk of type 2 diabetes: a systematic review and dose-response meta-analysis of more than 1.9 million individuals from 38 observational studies. Diabetes Care. 2015 Sep;38(9):1804–1812. 10.2337/dc15-0710 26294775

[3] Peng GS, Yin SJ Effect of the allelic variants of aldehyde dehydrogenase ALDH2^*^2 and alcohol dehydrogenase ADH1B^*^2 on blood acetaldehyde concentrations. Hum Genomics. 2009 Jan;3(2):121–127. 10.1186/1479-7364-3-2-121 19164089 PMC3525274

[4] Shin MJ, Cho Y, Davey Smith G Alcohol consumption, aldehyde dehydrogenase 2 gene polymorphisms, and cardiovascular health in Korea. Yonsei Med J. 2017 Jul;58(4):689–696. 10.3349/ymj.2017.58.4.689 28540979 PMC5447097

[5] Kawakita D, Oze I, Hosono S, Prognostic value of drinking status and aldehyde dehydrogenase 2 polymorphism in patients with head and neck squamous cell carcinoma. J Epidemiol. 2016 Jun 5;26(6):292–299. 10.2188/jea.JE20140240 26804037 PMC4884897

[6] Lim J, Lee JA, Cho HJ Association between alcohol drinking patterns and presence of impaired fasting glucose and diabetes mellitus among South Korean adults. J Epidemiol. 2017; In Press. 10.2188/jea.JE20170021 PMC5821688 29093361

[7] Schrieks IC, Heil AL, Hendriks HF, Mukamal KJ, Beulens JW The effect of alcohol consumption on insulin sensitivity and glycemic status: a systematic review and meta-analysis of intervention studies. Diabetes Care. 2015 Apr;38(4):723–732. 25805864 10.2337/dc14-1556

[8] Department of Health. UK Chief Medical Officers’ alcohol guidelines review: summary of the proposed new guidelines. 2016. http://www.gov.uk/government/uploads/system/uploads/attachment_data/file/489795/summary.pdf (accessed June 2017).

[9] Kromhout D, Spaaij CJ, de Goede J, Weggemans RM The 2015 Dutch food-based dietary guidelines. Eur J Clin Nutr. 2016 Aug;70(8):869–878. 10.1038/ejcn.2016.52 27049034 PMC5399142

